# Effectiveness of EEG-Biofeedback on Attentiveness, Working Memory and Quantitative Electroencephalography on Reading Disorder

**Published:** 2013

**Authors:** Elnaz Mosanezhad Jeddi, Mohammad Ali Nazari

**Affiliations:** 1 Psychologist, PhD Student, Department of Psychology, School of Psychology, The University of Tabriz, Tabriz, Iran.; 2 Neuroscientist, Assistant Professor, Department of Psychology, School of Psychology,The University of Tabriz, Tabriz, Iran.

**Keywords:** Coherence, Neurofeedback Training, Reading Disorder

## Abstract

**Objective:** Cognitive factors are the important correlates of reading disorder and their impairments are established in children with reading disorder. Neurofeedback as an intervention has been reported to be useful in improvement of cognitive deficits. The present study aimed to determine the effectiveness of this treatment on attentiveness and working memory and related electroencephalographic (EEG) changes in children with reading disorder.

**Methods:** In this single subject study, six children with reading disorder aged 8-10 years old completed twenty 30-minunt sessions of treatment. Continuous performance task, the digit span subscale of the 3^rd^ edition of Wechsler Intelligence Scale for Children (WISC-III) and quantitative electroencephalography were used to evaluate the changes at pre and post-treatment. The data were evaluated by visual inspection of the graph, the mean percentage improvement and signal detection measures.

**Results: **The results showed improvements in attention and working memory. Furthermore, EEG analysis did not show notable changes in the power of the targeted bands (delta, theta, and beta), rather the normalization of coherence was explicit in theta band at T3-T4, delta band at Cz-Fz, beta band at Cz-Fz, Cz-Pz and Cz-C4.

**Conclusions:** These significant changes in coherence are possible indications of the connectivity between frontal and posterior association and integration between sensory and motor areas that explain the improvements in attention and working memory.

**Declaration of interest:** None.

## Introduction

Reading disorder (RD) is characterized by serious reading difficulties based on intelligence, education, and age. Cognitive impairments are important correlates of functions in children with RD (1). Attention (2) and memory problems (3) have been associated with reading difficulties. There is evidence that working memory impairments have been related to some of academic problems in children with RD (3, 4). Baddeley’s working memory model assumes that growth in working memory underlies poor growth in reading skills (4). In one study of testing, Baddeley’s working memory model in children with RD Swanson (5) showed that they were inferior to skilled readers at all age levels. Moreover, the influence of working memory on measures of reading follows automatically with improvements in controlled attention (1). Dyslexic children show impairments of attention that is particularly important in reading performance (6). 

Focusing on the related brain structure, Blau et al. (7) observed under-activation of the superior temporal gyrus in people with dyslexia. The left superior temporal gyrus plays an important role in integrating auditory, perceptual and memory that is related to fluent reading (8). Lesions of the left supra-marginal gyrus (anterior to T3) are associated with deficits in spelling (9) and verbal memory (10). Dyslexics with planum temporale (PT) symmetry had phonological deficits in reading indicating PT involved in language processing (11). 

In addition to the brain structure, the electroencephalographic (EEG) activity in children with RD has some abnormalities (12). For example, Arns et al. (13) reported an increased slow activity (delta and theta) in the frontal and temporal regions on children with dyslexia which had problems in memory recognition test. The same dysfunction in the left superior temporal gyrus (T3) was also reported by Simos et al. (14). Mann et al. (15) showed that children with a diagnosis of attention deficit disorder had an increase in absolute power in the theta band, predominantly in the frontal regions and a greater increase in theta activity and in frontal and central regions during cognitive tasks, and also a decrease in beta activity in posterior and temporal regions with tasks requiring sustained attention. A similar results obtained by Chabot et al. (16), Clarke et al. (17), Lazzaro et al. (18). Beta activity (15-30 Hz) has also been related to cognitive activity. Townsend and Johnson (19) described a reduction in activity in the 15-20 Hz range preceding errors of omission.

To date, there are insufficient studies to evaluate the reliability of coherence differences and coherence studies on dyslexia and its related cognitive abilities show some controversies. Sklar et al. (20) showed higher intra-hemispheric coherence and lower inter-hemispheric coherence in children with dyslexia. Shiota et al. (21) in rest condition showed an increased inter and intra-hemispheric coherence in dyslexic children. Weiss and Muller (22) concluded a decreased coherence in people with dyslexia. Coherence studies on attention and working memory are also remarkable. Chabot et al. (23) reported that attention deficit was associated with inter-hemispheric and intra-hemispheric hyper coherence in frontal and central regions. Sarnthein et al. (24) reported that short-term/working memory was associated with theta phase synchrony between some frontal and posterior regions. An increased coherence between these regions during a semantic working memory task was reported by Haarmann and Cameron (25). Similarly, Kopp et al. (26) reported that memorization of words lists was associated with increased coherence in theta, beta and gamma frequency ranges. However, in another study by Onton et al. (27) no such association was observed between the frontal and posterior components. 

Neurofeedback training (NFT) as an intervention can be useful in treatment of different disorders via the regulation of EEG abnormalities. Improvements in cognitive functioning have been achieved as a result of this training. NFT to increase beta (15-18 Hz) and suppression of theta activity has been reported to improve attention deficits in some studies such as Mann et al. (15), Rossiter and La Vaque (28), Lubar and Lubar (29), Nash (30), and Nazari et al. (31). Consistent with these finding, Othmer et al. (32) in a research showed that this protocol could be improved cognitive skills such as attention, memory (digit span) and academic performance.

Fernandez et al. (33) developed a study on LD children who had high ratios of theta to alpha absolute power (theta/alpha). Positive behavioral changes and significant improvements in the cognitive performances were found which was not replicated in the control group. The behavioral changes continued for two years later. In a single case study, Thornton and Carmody (34) reported the positive effect of NFT on auditory memory and reading memory. Jacobs (35) also demonstrated a similar effect on learning, attention, social, and developmental deficits. Vernon et al. (36) reported positive effects of NFT on aspects of cognitive performance such as working memory. Using NFT on 10 dyslexic children, Breteler et al. (37) found a small improvement on their spelling skills, but no improvement was indicated in their reading abilities.

Based on the effectiveness of NFT for improving performance on cognitive processing in individuals with attention deficit disorder and normal subjects, we investigated the effectiveness of NFT to improve attention processing and working memory for children with RD.

## Materials and Methods

A multiple baseline single subject design was used to conduct the experiment. Six male dyslexic children with mean age of 8-10 years (mean = 9 and SD = 0.63 years) and average IQ of 101.5, (SD = 12.95) who had no history of brain injury, neuropsychological or psychiatric disorders were selected from Learning Disability Center of Tabriz. Diagnosis of reading disorder was confirmed by results on the Wechsler Intelligence Scale for children (WISC-III), and Reading Disability Checklist (38). 

Inclusive criteria were age 8-10, educational grade (grade 2 to 4), sex (male), and diagnosis of reading disorder. Exclusive criteria included children with mental retardation, epilepsy and emotional disorders. The study was fully explained to parents of each participant and then a written consent form was signed by the parents.

This type of design conceptually is used as few as two or three groups to test an intervention; therefore participants randomly were assigned into three groups. After a stable and/or predictable pattern of performance has been established for all baselines/groups (after 3 baselines), an intervention was introduced to group 1. In addition, after a stable and/or predictable pattern of performance has been established with the behavior to which intervention was applied, the intervention was applied to a second group (after 4 baselines). The baselines that intervention has not yet been applied to were continued and tracked without change.

Treatment involved 20 sessions of NFT twice or three times a week, each session lasting 30 minutes (min). Groups started the B phase with 2 weeks (4 sessions) interval between them. NFT was conducted by ProComp Infiniti Encoder and BioGraph Infiniti Software (version 5.1.3). The training protocol was set to decrease delta (1-4 HZ) and theta (4-8 HZ) activities and to increase activity in the beta (15-18 HZ) at T3 and F7 according to Rippon and Brunswick (39) and Arns et al. (13). 

For 12 sessions, all participants received the same NFT protocol only at T3. Then, for 8 remaining sessions, the NFT protocol was administered to both T3 and F7. The length of NFT for T3 was 20 min and 10 min for F7. Training sessions were separated into a 2-min baseline period (i.e. no feedback), 30-min feedback presentation and a 2-min baseline period again. Interactive video games were used as feedback for children. Before and after NFT, evaluations were administered. 

*The digit span subscale of WISC-III*

To evaluate the effectiveness of the NFT on the working memory, we administered the digit span subscale from the WISC-III. This measure has been used in previous studies as an index of working memory (40). The reliability coefficients was higher than 0.95 and the validity was high. The WISC-III had sufficient reliability and validity in Iranian students (41).

*Continuous Performance task (CPT-II)*

Focused attention was examined using a computerized Continuous Performance task (CPT-II). During sequential presentation of a series of letters, participants had to respond to all letters except “X”. Results reported on perceptual sensitivity (d´) as a measure of Signal Detection Theory (SDT). The measure of d´ provided information on how well the subject discriminates between targets and non-targets. It was calculated using the number of hit and false alarm rates. Hit showed that the subject succeeded to respond to non-targets (all other letters expect "X") and false alarm was targets ("X") that the subject erroneously responded to. Reliability coefficients for CPT-II were 0.75 that were adequate. Studies supported the acceptable validity of CPT-II for the research objectives (42).

*EEG recording and processing*

The EEG was recorded in an eyes-closed resting condition using 19 surface electrodes (Electro-Cap®) based on the international 10-20 system. The EEG was amplified by NeuroScan®. DC-50 Hz was filtered and recorded with link-ears reference at a sampling rate of 500 Hz. The impedance of electrodes was kept below 10 kΩ. The EEG signals were processed using NeuroGuide Delux® (version 2.3.8). Artifact rejection was based on both visual inspection and computerized selection. Epochs were also visually analyzed by an expert who determined the acceptance or rejection of each individual epoch accordingly. 

In total, 36-48 artifact free EEG epochs (2.5 sec) were selected for analysis. The EEG epoch time domain was then transformed into the frequency domain using a fast Fourier transformation (FFT) algorithm. The frequency bands were defined as followed: delta (1-4 Hz), theta (4-8 Hz), alpha(8-12 Hz), and beta (12-25 Hz). Absolute power, relative power, and coherence scores of electrodes were calculated for each frequency band. Using Neuro Guide normative database, all the power and coherence values were subsequently transformed into Z-scores.

*Statistical analysis*

The data were evaluated for the change in working memory by visual inspection of the graph and the Mean Percentage of Improvement (MPI). The change in attention was evaluated by perceptual sensitivity (d´) based on SDT. It was calculated using the formula: d´ = ABS (HR)-ABS (FAR), where HR = hit rate and FAR = false alarm rate (43).

## Results

*Working memory*

**Figure 1:**
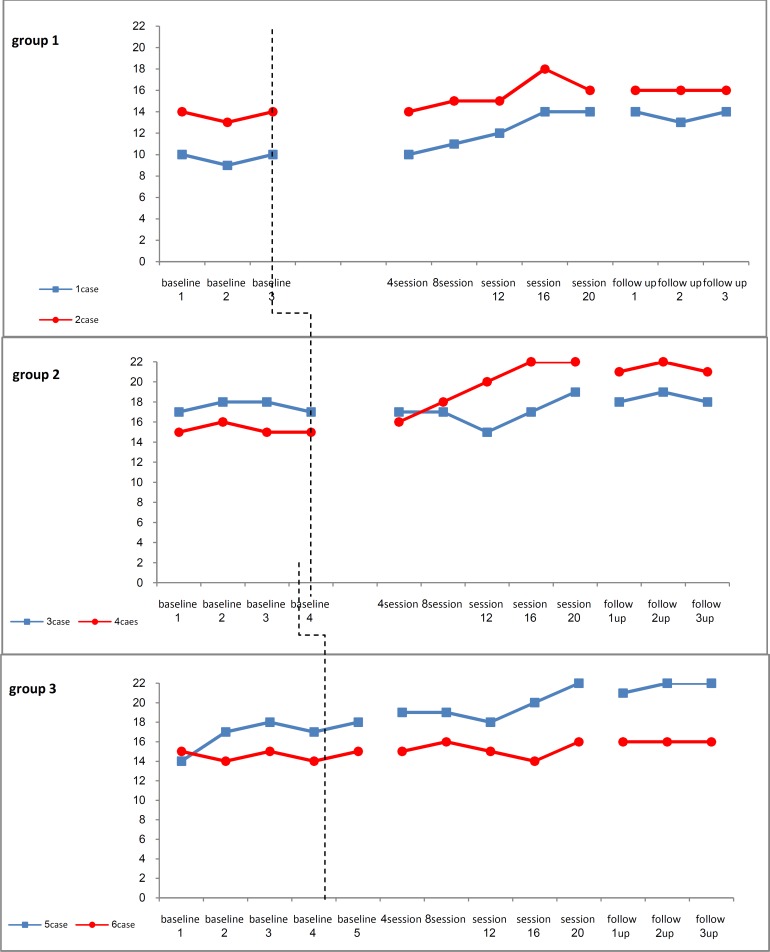
Working memory across baseline, treatment and post assessments conducted for all the three groups

Figure 1 shows the working memory scores at baseline, treatment, and post-treatment. Visual inspections show that baseline levels remained relatively stable and an increase in scores was shown only after the introduction of NFT. The participants showed the least amount of change after treatment. The value of mean for case 3 decreased after 12 sessions, but it increased thereafter.

Table 1 illustrates the mean, standard deviation (SD) and Mean Percentage Improvement (MPI) for all 6 participants at baseline, treatment, and post assessments for all participants. The level of working memory was increased in all but one subject (case 5). 

*Attention*

Table 2 shows the performance of participants at CPT-II and presents the measure of attentiveness or perceptual sensitivity (d´) for all subjects at pre and post–treatment phases. It could be shown that there was an improvement of d´ after NFT.

*Quantitative EEG (QEEG)*

**Figure 2:**
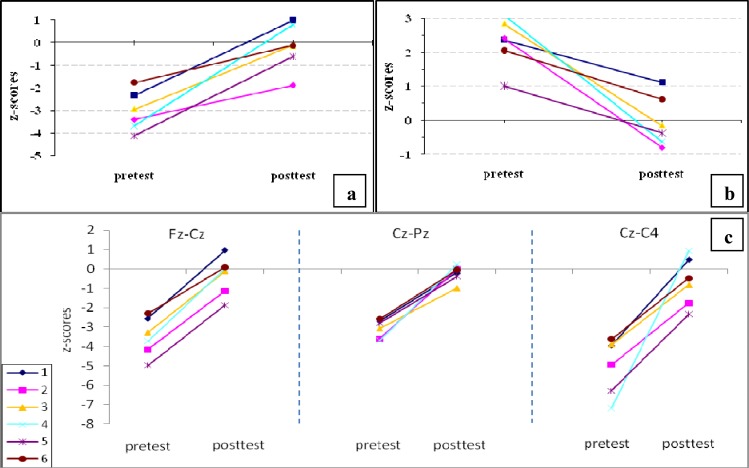
Pattern of z-scored coherence changes after NFT (a: coherence of delta band (Fz-Cz), b: coherence of theta band (T3-T4), c: coherence of beta band between different channels).

Results revealed no important changes in the power of the signal of any aimed frequency band and recording channels. However, coherence analysis showed an interesting change toward normalization in delta, theta, and beta bands after NFT. The coherence of delta band in Fz-Cz was up-trained from a lower-than-normal value to near normal. The pattern of changes in the theta band had a reduction of an abnormal hyper coherence toward near normal coherence between T3 and T4. The hypo coherence of beta band was approximated to normal in Fz-Cz, Cz-Pz and Cz-C4 (Figure 2).

## Discussion

Analysis of data revealed some changes in the working memory and attention by NFT in all the participants. The participants showed an improvement in the mean of working memory measures. In addition, post-treatment assessments showed that the changes in the participants remained durable and better compared to those of the baseline phase. Another change was in the measure of attentiveness (d´) at CPT-II performance. 

Different researches indicated that children with RD have deficits in working memory and attention as important correlates of reading ability (2, 3, 5, 6) and improvement of each of them could result in growth in reading skills (1).

Othmer et al. (32) reported the successful use of NFT for academic and cognitive improvements by decreasing theta and enhancing beta (15-18 Hz). Furthermore, some studies reported the efficacy of this protocol to improve attention deficits (28-30). 

**Table 1:** Working memory at baseline, treatment, post treatment

	**Mean**_A1_	**Mean**_B_	**Mean**_A2_	**SD**_A1_	**SD**_B_	**SD**_A2_	**MPI**
**Case 1**	9.67	12.20	13.67	0.58	1.79	0.58	20.81%
**Case 2**	13.67	15.60	16.00	0.58	1.52	0	12.43%
**Case 3**	17.50	17.00	18.33	0.58	1.41	0.58	-2.94%
**Case 4**	15.25	19.60	21.33	0.50	2.61	0.58	22.19%
**Case 5**	16.80	19.60	21.67	1.64	1.51	0.58	14.28%
**Case 6**	14.60	15.20	16.00	0.55	0.84	0	05.26%

A1: Baseline phase, B: Treatment phase, A2: Post treatment, SD: Standard deviation, MPI: Mean percentage improvement

**Table 2:** CPT-II performance measures for pre and post-treatment phases

**d´**	**Pre**	**Post**
**Case 1**	1.3792	1.5664
**Case 2**	0.9092	0.7306
**Case 3**	0.1497	1.9280
**Case 4**	2.9316	2.8310
**Case 5**	0.3865	1.5560
**Case 6**	1.1248	1.1388
**Mean**	1.1460	1.6250

In some single subject reports, Thornton and Carmody (34) reported improvements in reading, auditory memory, reading memory, and reading fluency after NFT. Some other studies supported these findings (35, 36, 44).

To explore the links between behavioral changes and EEG changes in our participants, EEG power and coherence were analyzed. The analysis showed no important change in power of the targeted bands (delta, theta, and beta) after NFT. However, coherence analysis showed interesting changes after the intervention. Inter hemispheric coherence normalization in theta at T3-T4 was observed. In addition, normalization of hypo coherence of the delta band at Cz-Fz, and of the beta band in Cz-Fz, Cz-Pz and Cz-C4 was found after treatment.

As a heterogeneous syndrome, RD has some abnormalities on the brain structure and the electroencephalographic (EEG) activity (22). For example, Sklar et al. (20) reported higher intra hemispheric coherence as well as lower inter hemispheric coherence in dyslexics during reading. Similarly, Leisman (45) found lower inter hemispheric and higher intra hemispheric coherence in dyslexics. However, Shiota et al. (21) showed high inter and intra-hemispheric EEG coherence values and Weiss and Muller (22) found a decreased coherence in children with dyslexia. About cognitive abilities, inter-hemispheric and intra-hemispheric hyper coherence in frontal and central regions was associated with attention deficits (23). An increased coherence in theta, beta and gamma frequency ranges was associated with semantic working memory tasks by Kopp et al. (26) and Haarmann and Cameron (25). Another study did not find this association (27). 

Different studies have suggested the left PT (around T3) as an important region in spelling and memory that was related to fluent reading (8, 46). The observed hyper coherence of theta band at T3-T4 may be an indication of abnormal symmetric function of the left/right temporal regions in dyslexics. The normalization of coherence resulting from NFT was the neuroplastic changes that can be effect on reading skills and correlated cognitive abilities. 

Moreover, some studies have pointed to the importance of connectivity between frontal and posterior association for working memory with different experiments on humans (24). For instance, Haarmann and Cameron (25) reported an increased coherence between these regions during the working memory tasks. Prefrontal and parietal neurons have increased activity during delay periods in delayed response tasks (25). Therefore, the interaction of posterior cortex, where sensory information is thought to be stored, and prefrontal cortex, where current information is maintained and continuously updated, mediates working memory processes (24). Nevertheless, the normalization (increase) of beta and delta coherence in Cz-Fz, Cz-Pz and Cz-C4 (in rest condition) as a result of our study might be a sign of integration between sensory and motor areas in the brain of the participants after NFT.

However, the small numbers of cases requires cautious interpretation, and additional replication would be advantageous. Future researches on this issue should include the larger samples. External validity of results from single subject research was enhanced through replication of the effects across different participants, different conditions, and/or different measure of the dependent variable.
